# A New Regulatory Mechanism for Kv7.2 Protein During Neuropathy: Enhanced Transport from the Soma to Axonal Terminals of Injured Sensory Neurons

**DOI:** 10.3389/fncel.2015.00470

**Published:** 2015-12-02

**Authors:** Elsa Cisneros, Carolina Roza, Nieka Jackson, José Antonio López-García

**Affiliations:** Departamento de Biología de Sistemas, Universidad de AlcaláAlcalá de Henares, Spain

**Keywords:** potassium channels, dorsal root ganglia, axonal endings, spared nerve injury, axotomy, axonal transport, vinblastine, colchicine

## Abstract

Kv7.2 channel expression has been reported to decrease in dorsal root ganglia (DRG) following the induction of a peripheral neuropathy while other experiments show that Kv7.2 accumulates in peripheral neuromas. The mechanisms underlying these novel expression patterns are poorly understood. Here we use immunofluorescence methods to analyze Kv7.2 protein expression changes in sensory neurons following peripheral axotomy and the potential role of axonal transport. Results indicate that DRG neurons express Kv7.2 in ~16% of neurons and that this number decreases by about 65% after axotomy. Damaged neurons were identified in DRG by application of the tracer Fluoro-ruby at the site of injury during surgery. Reduction of Kv7.2 expression was particularly strong in damaged neurons although some loss was also found in putative uninjured neurons. In parallel to the decrease in the soma of axotomized sensory neurons, Kv7.2 accumulated at neuromatose fiber endings. Blockade of axonal transport with either vinblastine (VLB) or colchicine (COL) abolished Kv7.2 redistribution in neuropathic animals. Channel distribution rearrangements did not occur following induction of inflammation in the hind paw. Behavioral tests indicate that protein rearrangements within sensory afferents are essential to the development of allodynia under neuropathic conditions. These results suggest that axotomy enhances axonal transport in injured sensory neurons, leading to a decrease of somatic expression of Kv7.2 protein and a concomitant accumulation in damaged fiber endings. Localized changes in channel expression patterns under pathological conditions may create novel opportunities for Kv7.2 channel openers to act as analgesics.

## Introduction

Kv7 (KCNQ) channels are a family of five voltage-gated K^+^ channel subunits (Kv7.1-Kv7.5) that yield the M-currents, crucial for controlling homeostasis to the point that mutations in their constituent subunits contribute to various diseases (Jentsch, 2000). In the nervous system, Kv7 channels play a key role in regulating excitability in many central and peripheral neuronal types,and their physiological function depends on their distribution in specialized subcellular domains such as axon initial segments, nodes and nerve terminals (Battefeld et al., 2014). Since M-currents are present at key locations in nociceptive pathways and their activation by selective compounds exerts antihyperalgesic actions in several models of pain, the interest in Kv7 modulation for analgesia has grown in later years (Rivera-Arconada et al., 2009). In most neurons, M-currents are produced by Kv7.2-Kv7.3 heteromers or Kv7.2 homomers (Brown and Passmore, 2009).

Recent studies have shown that Kv7.2 protein levels decrease in the dorsal root ganglia (DRG) from rats subjected to bone cancer or partial sciatic nerve ligation (PSNL) contributing to make DRG neurons more excitable (Rose et al., 2011; Zheng et al., 2013). In apparent contradiction, we have reported increased levels of Kv7.2 in the peripheral terminals of axotomized fibers from mice subjected to saphenous nerve axotomy (Roza et al., 2011) which may contribute to stabilize the terminals (Roza and Lopez-Garcia, 2008). At a behavioral level, channel opening using specific compounds like retigabine reduces hyperalgesia in animal models of neuropathy (Blackburn-Munro and Jensen, 2003).

These observations raise a number of basic questions that are presently unanswered. It is unknown whether the decrease of Kv7.2 expression in the DRG and its accumulation in the neuroma are concomitant events in a model of neuropathy involving axotomy. If this was the case, the mechanisms underlying channel rearrangement should be addressed. Channel expression in the different compartments of sensory neurons may depend on processes such as channel synthesis and degradation, transport and anchoring to the membranes. The decrease of Kv7.2 channels within the DRG following PSNL has been attributed to transcriptional repression (Rose et al., 2011), whereas the causes of accumulation within saphenous neuromas remain unexplored.

The working hypothesis proposed here is that the anterograde transport for Kv7.2 channels may be enhanced by axotomy, therefore contributing to reduce the content of Kv7.2 channels in cell somata while increasing its presence in terminals. Although this novel mechanism may coexist with others, we thought that its relevance could be estimated by blockade of axonal transport.

In order to test this hypothesis, we have used immunohistochemical techniques and a mouse model of neuropathy, which involves axotomy of two peripheral branches of the sciatic nerve (SNI). The expression pattern of Kv7.2 channels has not been studied in this model before. Moreover, this is the first time that the effects of neuropathy on Kv7.2 levels is investigated in DRG and neuroma from the same animals. We demonstrate channel redistribution following spared nerve injury (SNI) with reduced levels of Kv7.2 expression in DRG and increased levels in damaged terminals. Our experiments show that channel redistribution is seriously affected by disruption of axonal transport with vinblastine (VLB) or colchicine (COL) at non-neurotoxic doses (Dilley and Bove, 2008). We propose axonal transport as a mechanism able to modulate Kv7.2 levels in neuronal compartments following axotomy. This class of modulation has not been described for any Kv7 channels before.

Finally, we explore the impact of axonal transport on mechanical sensitivity. VLB and COL treatment had an allodynic effect similar to that produced by SNI. On the contrary, when either of these drugs were administered on SNI animals, allodynia was not developed. These results suggests that axonal transport has an important role in the structural and functional changes that follow nerve axotomy.

### Materials and Methods

#### Animals

Experiments were performed on adult female CD-1 mice weighing 25–45 g. Animals were maintained at 22°C on a 12 h light/dark cycle with *ad libitum* access to food and water. All procedures followed European legislation and were approved by the Animal Care Committee of the University of Alcalá.

#### Induction of Neuropathy and Fluoro-Ruby Application

During all the surgical procedures, the mice underwent deep anesthesia with 3–4% isoflurane (Laboratorios del Dr. Esteve SA, Barcelona, Spain) in 100% oxygen. For SNI (Shields et al., 2003) an incision was made in the skin on the lateral surface of the thigh, the sciatic terminal branches (sural, common peroneal and tibial nerves) were exposed and the common peroneal and the tibial nerves were ligated with 8/0 silk and sectioned distal to the ligature. The sectioned end was inserted into an approximately 3 mm long silicone tube (0.45 mm internal diameter) to prevent lateral innervation of surrounding tissue. The tube was tied in place with the ligature piece of silk and the incision was closed with 5/0 sutures. Animals were then housed and inspected periodically for infections or abnormal behavior. Tissue from these animals was collected at two time points in which mechanical allodynia starts to develop (5 days) and reaches a maximum (15 days; Bourquin et al., 2006).

In some animals 2 μl of a 5% Fluoro-ruby solution (Molecular Probes, Eugene, OR, USA; D-1817) were applied into the proximal stump of the sectioned nerves using a glass micropipette.

During sham surgery, sciatic nerve branches were exposed but untouched and Fluoro-ruby solution was applied onto the common peroneal and tibial nerves.

#### Blockade of Axonal Transport

VLB (Sigma-Aldrich, St. Louis, MO, USA; V1377) and COL (Sigma-Aldrich, C9754) were prepared in 0.9% saline and administered intraperitoneally at a dose of 1.25 and 1 mg/Kg respectively.

VLB was administered 15 days post-surgery for 4 consecutive days. In other group of animals VLB or COL were administered one day before SNI surgery and the following 5 consecutive days. Twenty-four hours after the last injection, DRG and sciatic nerves were harvested. Mice without axotomy and treated with VLB or COL were used as controls.

VLB and COL are microtubule-destabilizing agents which block axonal transport (Dziegielewska et al., 1976). Since VLB and COL bind tubulin in a reversible manner we administered them daily (Jordan and Wilson, 2004). We used doses proved to be effective at disrupting microtubules and not lethal when administered during the treatment period (Stock, 1967; Chang and But, 1986). Direct application of high doses of VLB or COL onto the nerves causes Wallerian degeneration starting at the site of application (Coleman, 2005). The concentrations of VLB and COL in the solutions injected to the animals in our experiments were 0.13 and 0.25 mM respectively. These concentrations are considered low and produce neither axonal degeneration nor effects on impulse conduction when applied directly onto the sciatic nerves (Dziegielewska et al., 1976; Fitzgerald et al., 1984; Kashiba et al., 1992; Dilley and Bove, 2008).

#### Induction of Peripheral Inflammation

Inflammation was induced by unilateral intraplantar injection of 50 μl of Complete Freund’s Adjuvant (CFA; Sigma-Aldrich, St. Louis, MO, USA; F5881). Paw edema was quantified by measuring dorso-plantar paw thickness before and after CFA injection. L4 DRG were collected 1 and 7 days after injection. During this time frame mechanical allodynia is strong and significant (Cobos et al., 2012). Control animals received an injection of 50 μl of sterile physiological saline.

#### Tissue Collection and Histochemistry

Mice were anesthetized with urethane and perfused intracardially with 4% PFA in 0.1 M phosphate buffer (PB). The sciatic nerve as well as the L4 DRG receiving a large input from the sciatic nerve were dissected and fixed by immersion in 4% PFA in PB for 1.5 or 1 h respectively.

Tissues were cryoprotected with 30% sucrose in PB, embedded in 7.5% gelatin/15% sucrose in PB, frozen and sectioned at 16 μm. In some cases, sciatic nerves were teased. The sections (or teased nerves) were incubated in blocking solution, 10% donkey serum in Tris-buffered saline (TBS), for 2 h and permeabilized in 0.5% Triton X-100 in TBS for 10 min. The samples were then incubated with primary antibodies, that were diluted in TBS, at room temperature for 18 h. Primary antibodies were rabbit anti-KCNQ2 (Abcam, Cambrige, UK; ab22897, 1:500), rabbit anti-PanNav (Chemicon, Billerica, MA, USA; AB5210, 1:500), goat anti-Peripherin Myelin P2 (PMP2; Santa Cruz Biotechnology, Inc., Dallas, TX, USA; sc-49304, 1:100) and rabbit anti-active Caspase-3 (Casp3; Abcam; ab2302, 1:100). Sections were washed in 0.2% Tween 20 in TBS and incubated with Alexa-Fluor 488-conjugated donkey anti-rabbit secondary antibody (Jackson ImmunoResearch Laboratories, Inc., West Grove, PA, USA; 1:500) at room temperature for 1.5 h. After washing in TBS, the tissue was counterstained with Dapi (Sigma-Aldrich; D9542, 0.1 μg/ml) to label the nuclei or with NeuroTrace Nissl stain (Molecular Probes; N-214, 1:200) to label the neuron cytoplasm or the axonal terminal axoplasm (Jung et al., 2012). Sections were mounted in Mowiol and analyzed with fluorescence microscopy (Olympus BX61, Tokyo, Japan). Fluoro-ruby dye was detected directly onto sectioned tissue 15 days post-surgery.

For each staining experiment, samples from all experimental conditions to be compared statistically were processed in parallel (using the same solutions, in the same environment, and with the same timing).

#### Image Analysis

All images were analyzed using Fiji software (Schindelin et al., 2012). To quantify positively stained cells or endbulbs, to perform diameter measurements of positively stained cells and to measure mean fluorescence intensity (MFI) of cells we set an intensity fluorescence threshold. This threshold value was calculated as the mean intensity of the background staining in a negative control with the secondary antibody plus five times its standard deviation. Thresholds were kept constant for all samples of each experiment. In DRG samples only neurons with nuclei were counted. To avoid counting of the same cell, sections were mounted consecutively into five slides, so that sections on the same slide were separated by at least 64 μm.

The percentage of positive neurons within the DRG from each animal was determined from counts of ~500 neurons. To quantify the total number of neurons DRG were co-stained with NeuroTrace Nissl stain that labels neuronal cytoplasm. The percentages obtained for animals from the same experimental group were averaged.

MFI was measured in Kv7.2(+) cells. The values obtained from all the cells in the same population and group were averaged.

The density of Kv7.2(+) structures within neuroma sections were determined by counting Kv7(+) structures positive for NeuroTrace Nissl stain (that also labels the axoplasm of axonal terminals) on higher magnification images (40× objective) of the central area of the neuroma.

Results are presented as mean ± standard error of the mean (SEM). Statistical analyses were performed with IBM SPSS statistics 22 software. Differences between mean values were analyzed with unpaired Student *t*-test with or without the Welch’s correction as appropriate. Diameter size distributions of neuron populations were compared by using Kolmogorov-Smirnov test. Significant difference was accepted for *p* < 0.05.

#### Behavioral Testing

Three groups of mice receiving either VLB or SNI or VLB + SNI treatments were tested for cutaneous mechanical allodynia. Three equivalent groups for COL were also used. Frequency of paw withdrawal was measured before and after each procedure adapting a previously reported method (Bourquin et al., 2006). Briefly, the sural territory of the hind paw was stimulated 10 times with a calibrated von Frey monofilament of 0.16 g (Stoelting, Woodvale, IL, USA) and the number of positive paw withdrawals was assessed. Response frequency was represented as mean ± SEM and the overall effect of each procedure was analyzed using a two-way analysis of variance (ANOVA) followed by a *post hoc* Tukey’s test. Significant difference was accepted for *p* < 0.05.

### Results

#### Kv7.2 Channel Expression in DRG Under Control, Neuropathic, and Inflammatory Conditions

As a positive control for the specificity of anti-Kv7.2 antibody, we performed immunofluorescence experiments in teased sensory fibers from naïve sciatic nerves. The labeling produced by the Kv7.2 antibody was similar to that of PanNav. Both stainings were flanked by the paranodal marker PMP2 (Supplementary Figure 1). These results are in agreement with previous reports about Kv7.2 channel localization (Devaux et al., 2004) indicating specific Kv7.2 labelling.

**Figure 1:**
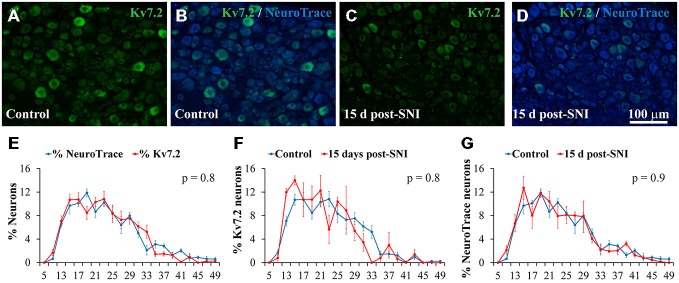
**Expression of Kv7.2 channel in the dorsal root ganglia (DRG) neurons of control and SNI animals 15 day post-surgery. (A−D)** Show cryostat sections of DRG from control and SNI animals respectively. Sections were immunostained with Kv7.2 antibody and counterstained with NeuroTrace to reveal all ganglia neurons. Scale bar in **(D)** applies to all images. **(E)** Graph showing the diameter distribution of neurons positive for Kv7.2 or NeuroTrace as indicated by color code. Graphs in **(F,G)** show diameter distribution of neurons labeled by Kv7.2 and NeuroTrace in control and SNI conditions. For each trace, the sum of percentages across all diameter intervals is 100%. The diameter was measured in at least 100 positively stained neurons in each DRG. Data points represent mean percentage values from three different ganglia. There were no significant differences between pairs of distributions as compared with the Kolmogorov-Smirnov test. The obtained *p* value is shown in the upper right corner of each graph.

Kv7.2 co-localized with NeuroTrace in sections of DRG, indicating selective expression in neurons, and accounted for 16.6 ± 2% of the DRG neuronal population of naïve adult mice (Table 1 and Figure 1A). Kv7.2 expressing neurons under naïve conditions had a diameter distribution almost identical to that of the general population of neurons of the ganglia (Figure 1E).

**Table 1:** **Percentage of Kv7.2(+) DRG neurons in control and SNI animals 15 days post-axotomy: effects of vinblastine**.

	Control	SNI	VLB Control	VLB SNI
% Kv7.2(+) neurons	16.6 ± 2.0	5.9 ± 0.7^**^	27.5 ± 3.5	29.5 ± 1.5 ^ns^
	*n* = 7	*n* = 6	*n* = 4	*n* = 7
% Kv7.2(+) neurons in FR(+) population	—	1.3 ± 0.2^**^	—	25.4 ± 4.4 ^ns^
		*n* = 4		*n* = 4
% Kv7.2(+) neurons in FR(−) population	—	9.5 ± 1.4^*^	—	31.2 ± 2.7 ^ns^
		*n* = 4		*n* = 4

FR, Fluoro-ruby (positive or negative staining); SNI, Spared nerve injury; VLB, Vinblastine treated. “n” stands for number of animals. Asterisk denote significant differences between control and SNI groups: (*p < 0.05, **p < 0.01). No differences were found between VLB Control and VLB SNI groups (ns, not significant).

Immunofluorescence analysis 15 days post-axotomy revealed a significant reduction in the percentage of Kv7.2(+) neurons that decreased by ~65% (Table 1; Figures 1A–D). These changes were not accompanied by significant alteration of neuronal density (1201 ± 79 and 1027 ± 81 neurons/mm^2^ in control and SNI conditions respectively; *n* = 6 mice each condition, *t*-test, *p* = 0.08). The percentage of neurons undergoing Casp3 activation was not significantly different between DRG from control and SNI animals (0% and 0.7 ± 0.4; *n* = 3 each condition). After SNI, no changes in diameter distribution were found for Kv7.2(+) neurons or the general population of DRG neurons (Figures 1F,G).

The percentage of Kv7.2(+) neurons 1 and 7 days after CFA injection were 15.9 ± 0.1% and 15.2 ± 1.1% respectively. These values were not significantly different from those obtained after saline injection (18.1 ± 2.5% and 16.3 ± 1.5% 1.7; *n* = 4 for 1 d experiments and *n* = 3 for 7 days experiments, *t*-test; see Supplementary Figure 2).

#### Kv7.2 Expression Predominantly Decreases in the Soma of Injured Neurons 15 Days After Axotomy

To check whether Kv7.2 loss was restricted to damaged neurons, Fluoro-ruby was applied into the proximal stump of sectioned nerves at the time of axotomy and immunostainig for Kv7.2 was performed 15 days post-axotomy. Fluoro-ruby is only taken up by injured axons and transported to the cell somata (Lozeron et al., 2004; Supplementary Figure 3). In lumbar DRG from SNI animals, axotomized and intact cells are intermingled. Therefore Fluoro-ruby tracing allowed us to differentiate injured (Fluoro-ruby positive) and uninjured (Fluoro-ruby negative) neurons in the same DRG.

**Figure 2:**
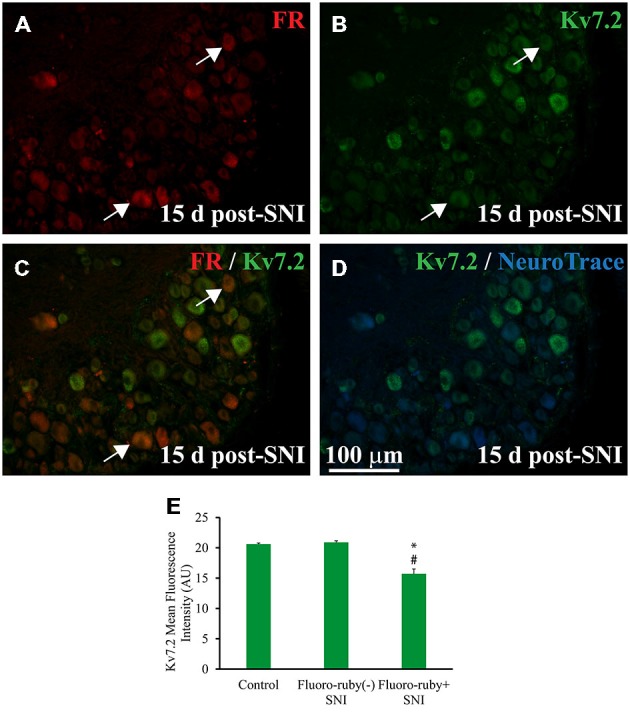
**Expression of Kv7.2 in Fluoro-ruby(+) DRG neurons 15 day post-surgery. (A)** Shows a representative section from the DRG obtained from an animal in which axotomized fibers (SNI) were labeled with Fluoro-ruby. **(B)** Shows same slide stained for Kv7.2.** (C)** is a merge of **(A,B)** in which arrows indicate the few neurons co-localizing Kv7.2 and Fluoro-ruby.** (D)** Shows Kv7.2 counterstained with NeuroTrace. **(E)** Graph shows mean fluorescence intensity (MFI) for Kv7.2 in DRG neurons from control and SNI animals. Note that MFI was significantly lower in Fluoro-ruby(+) from SNI animals compared to neurons in control DRG (*n* = 3; *t*-test, *p = 0.003) or Fluoro-ruby(-) neurons in SNI DRG (*n* = 3; *t-test*, ^#^*p* = 0.002). AU, arbitrary units. * or ^#^*p* < 0.05.

Fluoro-ruby traced neurons accounted for 20.5 ± 3.8% of DRG neurons (*n* = 4) of SNI animals. Kv7.2 staining fell by a much larger amount in Fluoro-ruby(+) neurons (~91%) than in Fluoro-ruby(−) neurons (~42%; Figures 2A–D and Table 1). The very few neurons that showed double labeling for Kv7.2 and Fluoro-ruby had significantly lower levels of Kv7.2 fluorescence than those un-labeled for Fluoro-ruby, or Kv7.2(+) cells in control DRG (Figure 2E).

#### Concomitant Kv7.2 Accumulation in Neuromatose Peripheral Endings 15 Days After Axotomy

In order to assess a potential concomitant increase in Kv7.2 expression in peripheral endings of damaged sensory fibers, we analyzed neuromatose fiber endings from animals that had been subjected to SNI treatment 15 days before and whose ganglia had been used for the previous experiment.

**Figure 3:**
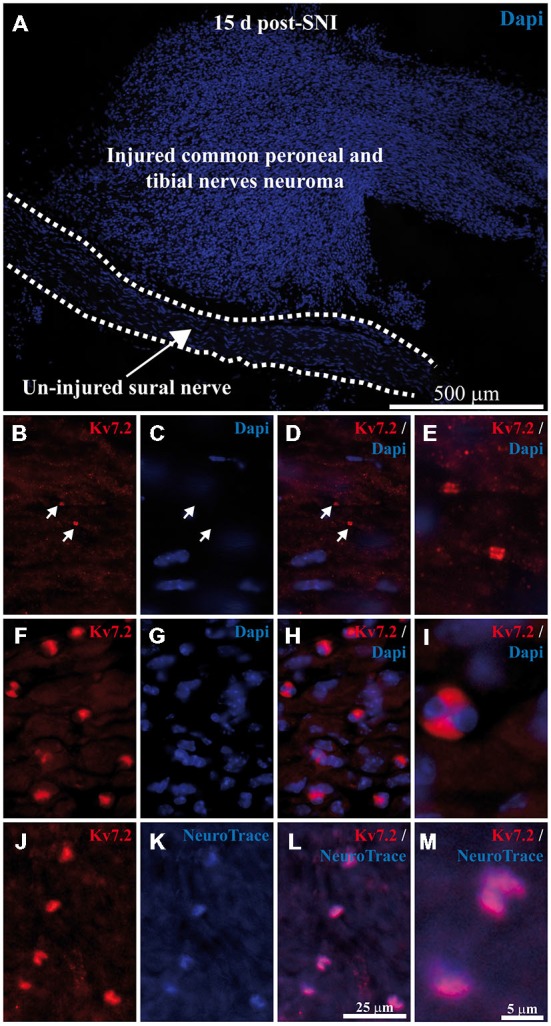
**Expression of Kv7.2 in neuromatose fiber endings 15 day post-axotomy. (A)** Sciatic nerve branches from SNI mice were sectioned and stained with Dapi. Injured fibers from common peroneal and tibial nerves (forming the neuroma) and un-injured fibers from sural nerve (delineated by dashed lines) are anatomically separated and can be readily identified. **(B–E)** images correspond to a magnification of the un-injured sural nerve. Sciatic nerves from SNI mice were sectioned, immunostained with Kv7.2 antibody and counterstained with Dapi to label cell nuclei. In sural intact branches labeling was found in normal nodes, indicated by white arrows in **(B–D)**, and was not associated with cell nuclei **(C–E)**. **(F–M)** images correspond to a magnification of injured common peroneal and tibial nerves. **(F–I)** Injured fibers show Kv7.2 in large structures which were surrounded by nuclei. **(J–M)** Show sciatic nerve sections from SNI mice immunostained with Kv7.2 and counterstained with NeuroTrace showing that Kv7.2(+) accumulations were localized in terminal axoplasm. Scale bar in **(M)** applies to **(E,I)** images. Scale bar in **(L)** applies to the rest of images.

In contrast to the ganglia, where injured and intact cells are intermingled, axotomized and intact fibers are anatomically separated and can be readily identified in the periphery (Figure 3A). Whereas the intact sural nerve only showed normal Kv7.2 nodal staining (Figures 3B–E) we found abnormal accumulations of Kv7.2 in peroneal and tibial fibers within the neuroma (Figures 3F–M).

Neuromatose accumulations of Kv7.2 were larger than those of normal nodes (4.2 ± 0.2 μm vs. 1.7 ± 0.2 μm, *p* < 0.0001, compare Figures 3E,I). In addition, neuromatose accumulations were surrounded by nuclei, probably from Schwann cells, (Figures 3F–I). These accumulations showed a complete co-localization with NeuroTrace (Figures 3J–M) which stains the neuronal cytoplasm as well as terminal axoplasm (Jung et al., 2012).

These Kv7.2(+) structures were abundant in the distal side of neuromas (30 ± 4.7 structures/0.1 mm^2^, *n* = 3 mice) and barely preset in the proximal side where fiber organization is better conserved (Supplementary Figure 4). This pattern of staining was not present in the intact sural nerve of experimental animals (Figures 3B–E), or in the sciatic branches from naïve animals (not shown).

**Figure 4:**
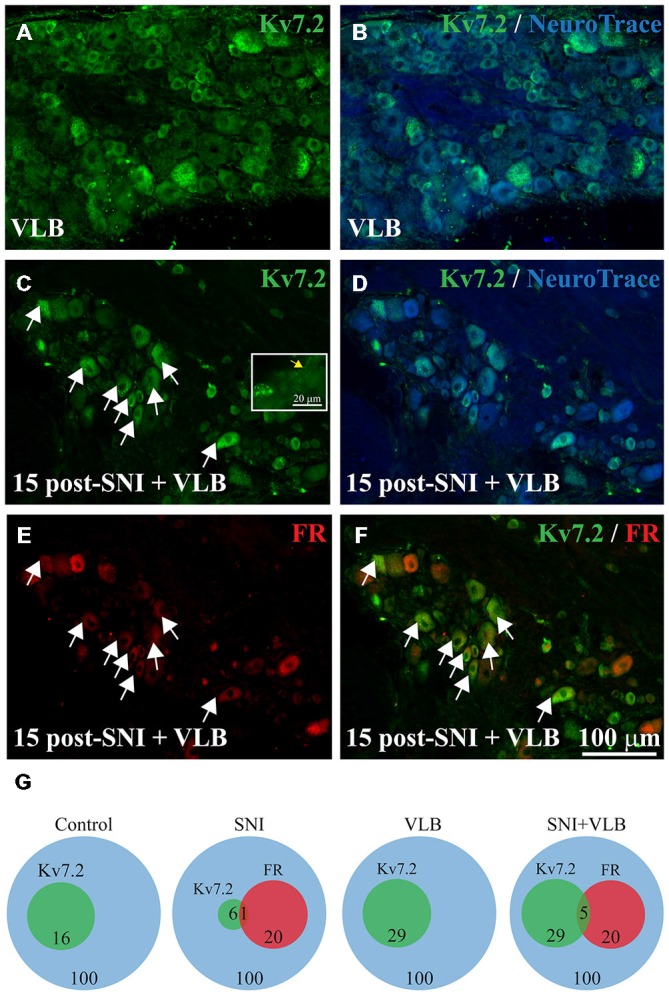
**Expression of Kv7.2 in the DRG from mice treated with Vinblastine (VLB) since day 15 post-surgery**. Sections of DRG from VLB-treated control **(A,B)** and VLB-treated SNI mice **(C–F)** were immunostained with Kv7.2 antibody and counterstained with NeuroTrace. The percentage of Kv7.2(+) neurons was similar in both samples. Inset in **(C)** shows Kv7.2 labeling in axons of DRG neurons (yellow arrow), suggesting an effective blockade of axonal transport. **(E)** Shows DRG neurons traced with Fluoro-ruby. The merge of **(C,E)** is shown in **(F)** where a large proportion of Fluoro-ruby(+) and Kv7.2(+) neurons can be appreciated after VLB treatment. Scale bar in **(F)** applies to all images. **(G)** Venn diagrams summarizing the percentage of Kv7.2(+) neurons in the DRG from non-axotomized or SNI mice that were untreated or treated with VLB. FR, Fluoro-ruby positive neurons.

#### Role of Axonal Transport in the Redistribution of Kv7.2 After Neuroma formation

A group of animals with a developed neuroma (15 days after axotomy) and a control non-axotomized group were treated with VLB to block axonal transport. Following VLB treatment, Kv7.2 labeling was detected within the axons leaving the DRG in both control and axotomized animals suggesting an effective blockade of the transport (Figure 4C inset).

Quantitative analysis as shown in Table 1 indicates that VLB treatment eliminates the difference in DRG labeling of Kv7.2 produced by axotomy, such that after VLB the percentage of neurons labeled for Kv7.2 in injured animals was very similar to that of uninjured animals (Figures 4A–D and Table 1).

VLB produced a ~1.7 fold increase in the percentage of Kv7.2(+) neurons in non-axotomized animals. This small but significant increase suggests a physiological transport of the channel (*t*-test, *p* = 0.02, Table 1). In SNI animals, VLB produced a ~5 fold increase in the percentage of Kv7(+) neurons (*t-test*, *p* < 0.0001, Table 1). This higher increase in the Kv7.2(+) neurons suggests an enhanced transport after axotomy.

The effects of VLB on Kv7.2 expression were also examined in experiments with Fluoro-ruby labeling of axotomized fibers. The percentage of Fluoro-ruby(+) neurons in these experiments was 22.8 ± 2.5% (*n* = 4; Figure 4E). The transport of Fluoro-ruby from the sectioned nerve end to the soma within the DRG was not affected by VLB, since it was administered 15 days post-axotomy, time at which Fluoro-ruby had reached the DRG already.

Following VLB treatment, Kv7.2 was present in 25.4 ± 4.4% of Fluoro-ruby(+) neurons, which represents a ~20 fold increase over injured animals without VLB. In addition, Kv7.2 staining was present in 31.21 ± 2.7% of Fluoro-ruby(-) neurons of the ganglia, a percentage ~4 fold higher than that observed in the corresponding control. After VBL treatment the percentage of Kv7(+) neurons was similar for Fluoro-ruby(+) and Fluoro-ruby(−) populations (*t*-test, *p* = 0.3, Figures 4C–F and Table 1). Results are summarized in Venn diagrams of Figure 4G.

Under the present conditions, VLB treatment did not change the expression pattern of Kv7.2 in the periphery. Channel accumulations co-localizing with NeuroTrace at neuromatose endings were observed at the same density after VLB treatment (18 ± 2.5 structures/0.1 mm^2^, *n* = 3 mice vs. 30 ± 4.7 structures/0.1 mm^2^ in un-treated 15 days SNI, *n* = 3 mice; *p* = 0.10). In the sciatic nerve from non-axotomized animals and in the uninjured sural nerve from SNI mice, Kv7.2 displayed the normal nodal staining.

#### Role of Axonal Transport in the Redistribution of Kv7.2 in Sensory Neurons during neuroma development

**Figure 5:**
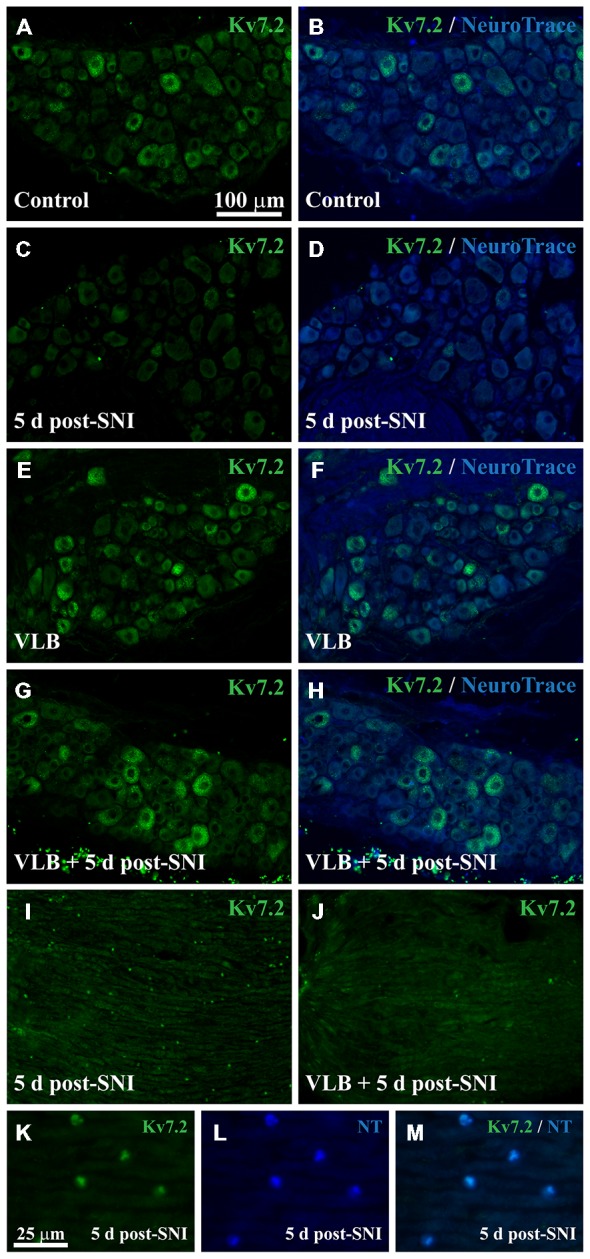
**Expression of Kv7.2 in the DRG and neuromas from mice treated with VLB during neuroma development**. **(A–H)** Show images of representative ganglia stained with Kv7.2 and counterstained with NeuroTrace from each experimental condition as labeled. Kv7.2 labelled neurons decrease within the DRG 5 days after axotomy. VLB administration before and during neuroma development prevents the reduction of Kv7.2 labeled neurons. **(I)** Shows a 5 day neuroma in which Kv7.2 accumulations are evident. **(K–M)** are a magnification of **(I)** to show that Kv7.2 structures are co-labeled with NeuroTrace. **(J)** Show a 5 day neuroma from an animal treated with VLB before and during neuroma formation. Note that Kv7.2(+) structures are virtually absent. Scale bar in **(A)** applies to **(B–J)** images. Scale bar in **(K)** applies to **(L,M)** images.

We asked whether axotomy produces a short-term Kv7.2 redistribution in sensory neurons. Immunofluorescence analysis 5 days post-surgery revealed a significant reduction in the percentage of Kv7.2(+) neurons in the DRG from SNI animals (~60%, Table 2) and the presence of Kv7.2 accumulations in the injured tibial and common peroneal fibers within the neuroma (Figure 5). These structures co-localized with NeuroTrace and where not found in the intact sural nerve, which only showed normal nodes (not shown). These results are similar to those obtained 15 days post-surgery.

**Table 2:** **Percentage of Kv7.2(+) DRG neurons in control and SNI animals 5 days post-axotomy: effects of vinblastine and colchicine**.

	Control	SNI	VLB Control	VLB SNI
% Kv7.2(+) neurons	13.9 ± 1.5	5.5 ± 0.6^*^	33.1 ± 3.4	29.2 ± 1.7 ^ns^
	*n* = 3	*n* = 3	*n* = 3	*n* = 3
	Control	SNI	COL Control	COL SNI
% Kv7.2(+) neurons	16.2 ± 2.0	4.5 ± 1.4^**^	14.0 ± 0.2	13.7 ± 1.4 ^ns^
	*n* = 3	*n* = 3	*n* = 3	*n* = 3

COL, Colchicine treated; SNI, Spared nerve injury; VLB, Vinblastine treated. “n” stands for number of animals. Asterisk denote significant differences between control and SNI groups: (*p < 0.05, **p < 0.01). No differences were found between Control treated with VLB and COL and corresponding SNI groups (ns, not significant).

VBL was applied to a group of animals for 6 days starting 1day pre-axotomy and to a control group without surgery for the same time lapse. To determine whether VLB produced neurotoxicity we analyzed Casp3 activation in the sensory neurons of mice receiving VLB, SNI o VLB + SNI. Active Casp3 was not detectable in the sensory neurons of any group (*n* = 3 mice each group; Supplementary Figure 5).

Under VLB treatment, the percentage of Kv7.2(+) neurons in the DRG was not significantly different between SNI and control animals. VLB produced a ~5 fold increase in the percentage of Kv7(+) neurons 5 days post-axotomy (*t*-test, *p* = 0.002, Table 2). This result is similar to that observed when VLB was administered from day 15 post-axotomy onwards. In the VLB-treated control DRG the percentage of Kv7.2(+) neurons increased ~2.4 fold (*t-test*, *p* = 0.05, Table 2), indicating again that axonal transport is enhanced following axotomy (Figures 5A–H).

VLB administration before axotomy changed the expression pattern of Kv7.2 in the periphery. Kv7.2 accumulations were virtually absent within the neuromas from VLB-treated animals, when compared to those from VLB-untreated SNI mice (6 ± 0.2 and 28 ± 0.7 structures/0.1 mm^2^ respectively, *n* = 3 mice each condition; *t*-test, *p* = 0.001; Figures 5I–M).

To confirm the role of axonal transport in Kv7.2 redistribution following axotomy we analyzed the effect of another transport blocker drug, COL. COL was administered following the same time course described above for VLB and DRG and neuromas were analyzed by immunofluorescence. COL treatment abolished the difference in the percentage of Kv7.2(+) neurons between control and SNI animals (Table 2). COL treatment did not changed the percentage of Kv7.2(+) neurons in control DRG (Table 2; *t*-test, *p* = 0.4).

In the periphery, the density of Kv7.2(+) structures significantly decreased within the neuromas from animals treated with COL, when compared with neuromas from un-treated animals (4.2 ± 0.4 and 28 ± 0.7 structures/0.1 mm^2^ respectively, *n* = 3 mice each condition; *t-test*, *p* = 0.002, Supplementary Figure 6).

#### Axonal Transport and Nociceptive Behaviors

In order to obtain clues about the possible functional meaning of channel transport and subsequent rearrangements, we tested sensitivity to low threshold mechanical stimuli in three groups of animals receiving either VLB or SNI or VLB + SNI treatments. Baseline values were obtained for all groups before any other procedures at day 0. Immediately afterwards still in day 0, VLB was administered to animals of VLB and VLB + SNI groups which received daily doses until the end of the experiment. At day 1, axotomy was performed in animals of the SNI and VLB + SNI groups. Mechanical responses were tested again at day 6.

**Figure 6:**
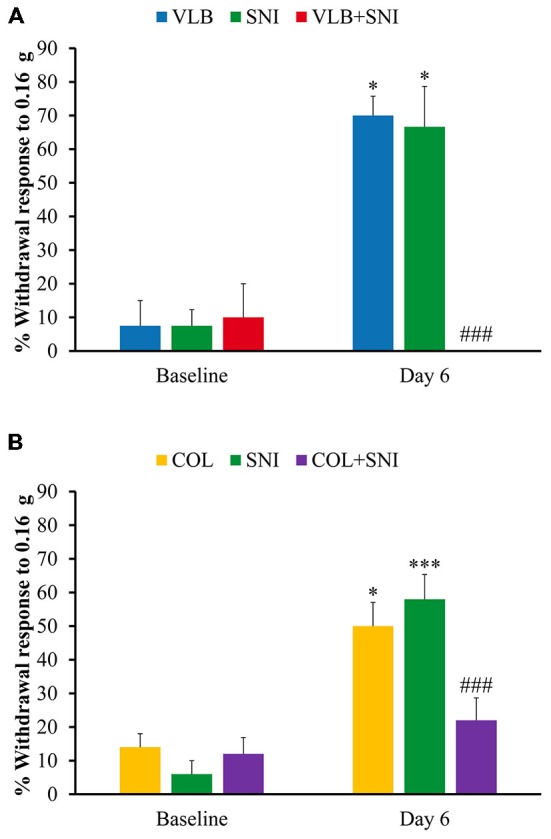
**Effect of blockade of axonal transport on mechanical sensitivity**. Graphs show behavioral effects of VLB **(A)** and Colchicine (COL) **(B)** on control and after 6 days of drug treatment. Bars represent percentage of withdrawals to a set of 10 stimuli applied with a Von Fray filament (0.16 g) to the plantar surface of the hind paw (*n* = 4 for VLB and *n* = 5 for COL, ANOVA with *post hoc* Tukey’s test; *denote differences with corresponding baseline values; ^#^denote differences between VLB- or COL-treated SNI animals and other conditions at day 6 of treatment).

The results of this experiment are shown in Figure 6A. As expected, allodynic effects were obvious in the SNI group. Animals receiving VLB alone did develop allodynia to a similar degree than axotomized animals. However, VLB administered to axotomized animals produced a complete reversion of allodynia.

To confirm that behavioral changes observed after VLB treatment were due to axonal transport blockade, we measured mechanical response in animals receiving COL, SNI and COL + SNI in the same conditions described above. COL alone produced an increase in mechanical responses similar to that observed in SNI animals. On the contrary, COL treatment had an analgesic effect in SNI animals, although milder than that produced by VLB (Figure 6B).

### Discussion

Consistent with previous reports, we find that Kv7.2 is expressed in approximately 16% of the neuronal population of mice DRG. Under our conditions, analysis of diameter distribution indicates that Kv7.2 is present in sensory neurons of all sizes and no enrichment in small or large neurons was detected (Passmore et al., 2003; Rose et al., 2011; King and Scherer, 2012; Zheng et al., 2013).

The present results show a significant decrease of Kv7.2(+) neurons in the DRG of animals that had undergone an SNI procedure. The decrease is not due to neuronal loss, since neuronal density remains unchanged and the percentage of active Casp3 is anecdotal. Additionally, the decrease is not associated to small or large cells, but rather it distributes uniformly with neuronal diameter. These results are consistent with previous studies showing a decrease of Kv7.2 levels in the DRG of rats subjected to bone cancer and peripheral sciatic nerve ligation (Rose et al., 2011; Zheng et al., 2013) indicating that the Kv7.2 decrease in the DRG is a general mechanism triggered by peripheral nerve damage and shared by different species. Here we show that other pain-producing conditions, such as peripheral inflammation, do not produce any change in somatic levels of Kv7.2 channels.

The present results show that most ganglion cells decreasing their levels of Kv7.2 expression correspond to axotomized fibers. According to our results, Kv7.2 labeling is virtually lost in fibers marked with Fluoro-ruby at the time of the lesion (~91% reduction). However, uninjured neurons within the DRG of SNI treated animals appear to contribute towards Kv7.2 decrease, although to a lesser extent (~42% reduction). In this regard, it has to be considered that whereas axotomized neurons were unequivocally identified by Fluoro-ruby tracing, some axotomized neurons perhaps did not incorporate Fluoro-ruby. A study using ATF3 to mark injured cells has shown that common peroneal and tibial nerves account for 35% of fibers contributing to the L4 ganglion (Laedermann et al., 2014), whereas here we report only 20% of neurons with Fluoro-ruby. Consequently, the contribution of intact neurons could have been overestimated. Extrapolating our results to a scenario where 35% of DRG neurons were injured, we could still find a percentage of apparently intact neurons with reduced Kv7.2 expression. In this context, we cannot propose this phenomenon to affect selectively to damaged neurons. However, it is possible that modulation of Kv7.2 in intact neurons is due to their capacity to respond to soluble molecules secreted by neighboring injured neurons (Shinder et al., 1999).

Concomitant to the decrease of Kv7.2 levels in the DRG, an abnormal accumulation occurred in axotomized peroneal and tibial fibers within the neuroma, while the intact sural nerve displayed the normal nodal Kv7.2 staining (Devaux et al., 2004; Roza et al., 2011). Previously, we reported the accumulation of Kv7.2 in abnormal patterns at nodes of axotomized saphenous fibers (Roza et al., 2011). In addition to these abnormal patterns, here we describe for the first time the accumulation of the channel in neural structures surrounded by nuclei, which according to previous descriptions could be neuromatose endbulbs, sites which may work as ectopic sensors and generators of spontaneous activity (Fried et al., 1991; Devor et al., 1993). Nerve injury, then, triggers a process of redistribution of Kv7.2 channels that predominantly affects to injured neurons.

The major finding of the present work is to show the contribution of axonal transport to Kv7.2 channel rearrangement. To check this point we have treated naïve and SNI mice with VLB or COL. These two agents effectively block axoplasmic anterograde and retrograde transport (Dziegielewska et al., 1976). On the basis of available data, we have used concentrations that do not produce axonal degeneration neither effects on impulse conduction that are seen when high doses of the blockers are directly applied onto the sciatic nerves (Dziegielewska et al., 1976; Fitzgerald et al., 1984; Kashiba et al., 1992; Dilley and Bove, 2008). Moreover, we have not detected activation of Casp3 within the DRG of control or SNI animals treated with VLB. All this indicates that the effects of VLB and COL are due to their axon transport blocking properties.

In control animals, administration of VLB increased the percentage of Kv7.2(+) neurons by ~1.7 fold. This increase produced by VLB may reflect basal axonal transport working under physiological conditions. However, administration of COL did not affect channel levels in control animals (Tables 1, 1, 2) suggesting that either COL is less effective than VLB or that VLB may have some nonspecific effects. Regardless of differences between the compounds, both of them were able to dampen the effects of SNI on Kv7.2 loss in DRG when applied after neuroma formation or during its development.

When VLB was applied 15 days post-SNI, the percentage of Kv7.2(+) neurons increased up to ~20 fold within the population of Fluoro-ruby(+) neurons from SNI animals indicating that axotomized fibers have an enhanced transport. Most importantly, the treatment of neuropathic mice with VLB restored the levels of Kv7.2 injured neurons within the DRG (Table 1). On the other hand, accumulations of Kv7.2 within endbulbs were retained. In this set of experiments, VLB was administered to SNI animals once neuromas and Kv7.2 accumulations had already formed. As VLB blocks retrograde transport as well, we believe that under these conditions the channel was retained at axonal terminals. In contrast, peripheral accumulations of Kv7.2 were prevented by VLB and COL when these were applied prior and during neuroma development. Interestingly, blockers of axonal transport applied during the development of the neuroma did reverse the decrease of Kv7.2 expression in DRG as well. Together these results suggest that axonal transport may be a major mechanism operating after axotomy to produce channel distribution rearrangements in sensory neurons.

The levels of a protein in a particular cell compartment at a fixed time is the result of the production (rate of transcription and translation), transport to the target compartment (by molecular motors as kinesins), fixation to target compartments through linker proteins (as Ankyrin-G in the case of Kv7.2) and recycling/degradation processes. Therefore, Kv7.2 expression pattern in axotomized sensory neurons could be mediated by different mechanism. In a model of neuropathy involving nerve ligation (PSNL) but not axotomy, Kv7.2 decrease within the DRG has been attributed to transcriptional repression (Rose et al., 2011). Under the present experimental conditions and in our hands, the results obtained are not consistent with a major role for this mechanism suggesting the implication of different mechanisms under different lesion types.

There is evidence for a role of axonal transport in the distribution of other ionic channels after injury. Nav1.8 channel display a similar redistribution after chronic constriction injury, diminishing in the DRG and accumulating along the sciatic nerve (Novakovic et al., 1998). In models of peripheral inflammation, accumulation of Nav1.8 in the sciatic nerve depends on the interaction with the motor protein KIF5B (Su et al., 2013). The molecules that promote Kv7.2 transport to the periphery are unknown, but KIF5 proteins are possible candidates since they have been shown to interact with potassium channels (Xu et al., 2010).

Redistribution of proteins such as Nav or Kv channels, as a consequence of changes in axonal transport might be one important factor conditioning the development of pain signs following peripheral nerve damage. Here we show that the overall blockade of axonal transport with VLB or COL has different effects on axotomized and control animals.The present data show that VLB or COL applied to control non-axotomized animals produces mechanical allodynia similar to that produced by SNI treatment. Previous data showed that local application of VLB and COL to the sciatic nerve produces an increase in mechanical sensitivity probably caused by ectopic accumulation of mechanically sensitive ion channels, nerve growth factors or voltage-gated channels proximal to the site of transport blockade (Dilley and Bove, 2008; Dilley et al., 2013).

VLB and COL administered before and during neuroma development to our SNI animals exerted anti-allodynic effects. In line with this, it has been shown that VLB reduces ectopic discharges in injured nerves (Devor and Govrin Lippmann, 1983) suggesting that the components mediating mechanical sensivity need to be transported following axotomy. In addition, VLB and COL have been reported to exert anti-inflammatory effects due to their anti-proliferative actions (Dilley and Bove, 2008). This could have contributed towards the anti-allodinic effet of the blockers.

In the absence of a more specific tool to block Kv7 channel transport only, these experiments show a clear contribution of axonal transport to the estructural and functional changes that occur in sensory afferents after injury.

The specific pathophysiological significance of Kv7.2 decrease in the DRG and accumulation in the injured fibers is difficult to anticipate. Due to the properties of Kv7 channels, increased expression should exert stabilizing effects on membranes hyperpolarizing the resting potential and decreasing excitability. This should increase the threshold for the firing of action potentials initiated by Nav channels (Battefeld et al., 2014). Experimental evidence obtained in peripheral damaged fibers do confirm this interpretation (Roza and Lopez-Garcia, 2008). Therefore we interpret that the increased expression of Kv7.2 in neuromas may work as a homeostatic mechanism compensating, at least in part, for an incresed excitability caused by accumulation of Nav channels, transductor proteins and growth factors. The overexpression of Kv7 channels in neuromas may decrease their responsiveness to natural stimuli and the chances to originating ectopic spontaneous discharges.

In contrast, a decreased expression of Kv7.2 channels should cause the opposite effects, that is depolarization and increased excitability. In agreement, experimental data obtained by King et al. (2014) show that a selective decreased expression of Kv7.2 renders DRG neurons more excitable. This in turn may contribute towards pain symptoms caused by neuropathy such as spontaneous pain.

### Author Contributions

EC has contributed to the design of experiments, carried out most of the experimental work, performed the analysis of results and wrote the manuscript. CR has contributed towards the experiments and discussion of results. NJ has contributed to behavioral experiments. JAL has contributed to the design of the experiments and the writing of the manuscript.

### Funding

This work was supported by MINECO Spain (BFU 2012-37905). EC is supported by MINECO.

### Conflict of Interest Statement

The authors declare that the research was conducted in the absence of any commercial or financial relationships that could be construed as a potential conflict of interest.
