# Supplementary material for: A New Regulatory Mechanism for Kv7.2 Protein During Neuropathy: Enhanced Transport from the Soma to Axonal Terminals of Injured Sensory Neurons
Source: Front Cell Neurosci. 2015 Dec 2;9:470. doi: 10.3389/fncel.2015.00470 (PMC4667099; doi:10.3389/fncel.2015.00470)
Supplement: Supplementary file 1 [file DataSheet_1.docx]

Supplementary Material

A new regulatory mechanism for Kv7.2 protein during neuropathy: enhanced transport from the soma to the axonal terminals of injured sensory neurons

Elsa Cisneros, Carolina Roza, Nieka Jackson, José Antonio López-García*

*** Correspondence:** [josea.lopez@uah.es](mailto:josea.lopez@uah.es).

## Supplementary Figures


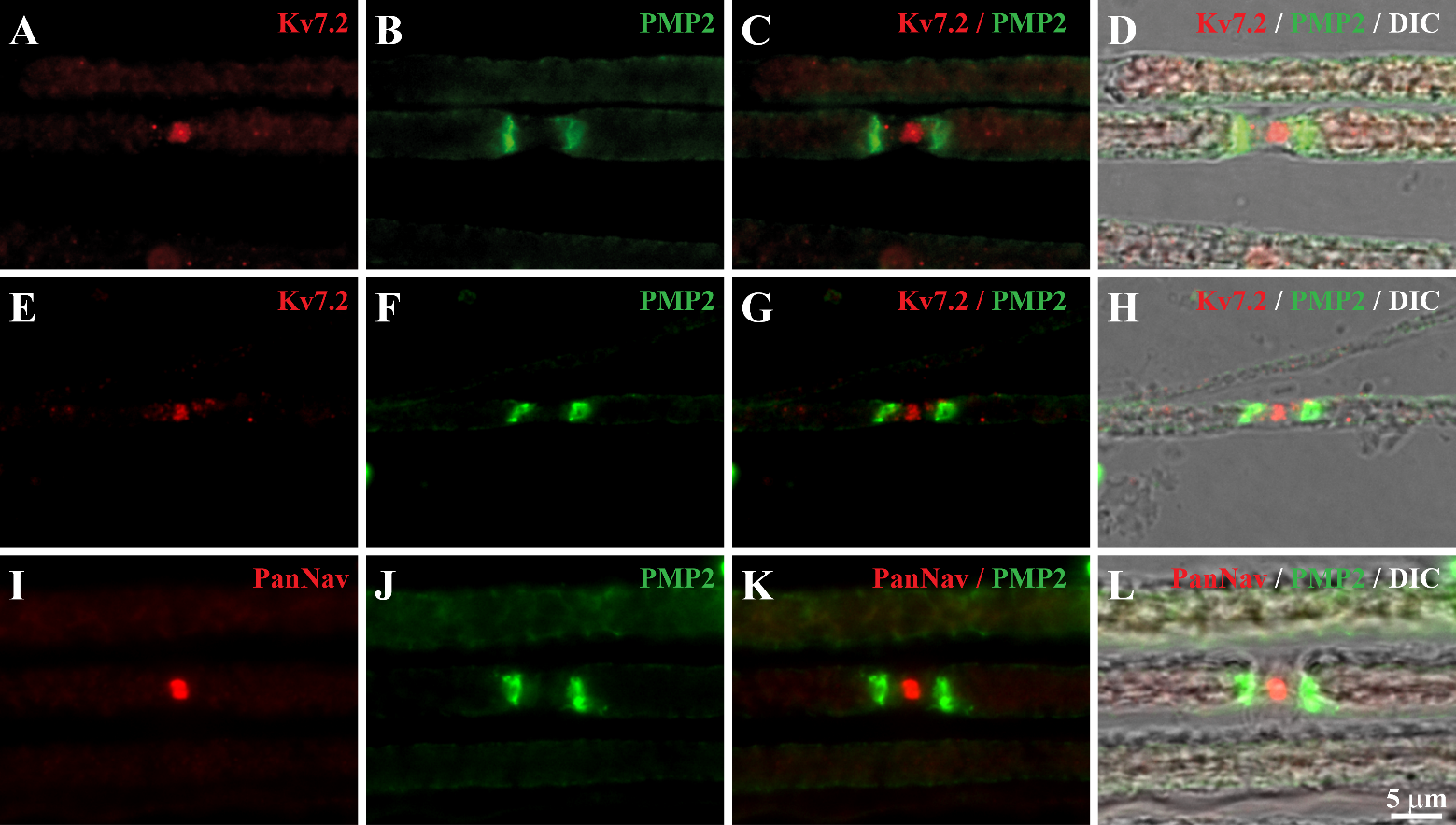


**Supplementary Figure 1.** Validation of anti-Kv7.2 antibody. Teased fibers were immunostained with antibodies against Kv7.2 and PMP2 (A-H) or PanNav and PMP2 (I-L) as labeled in images. Scale in (L) applies to all images. Kv7.2 staining is flanked by the paranodal marker PMP2. The same expression pattern was observed for PanNav, which marks nodes. DIC: Differential interference contrast.


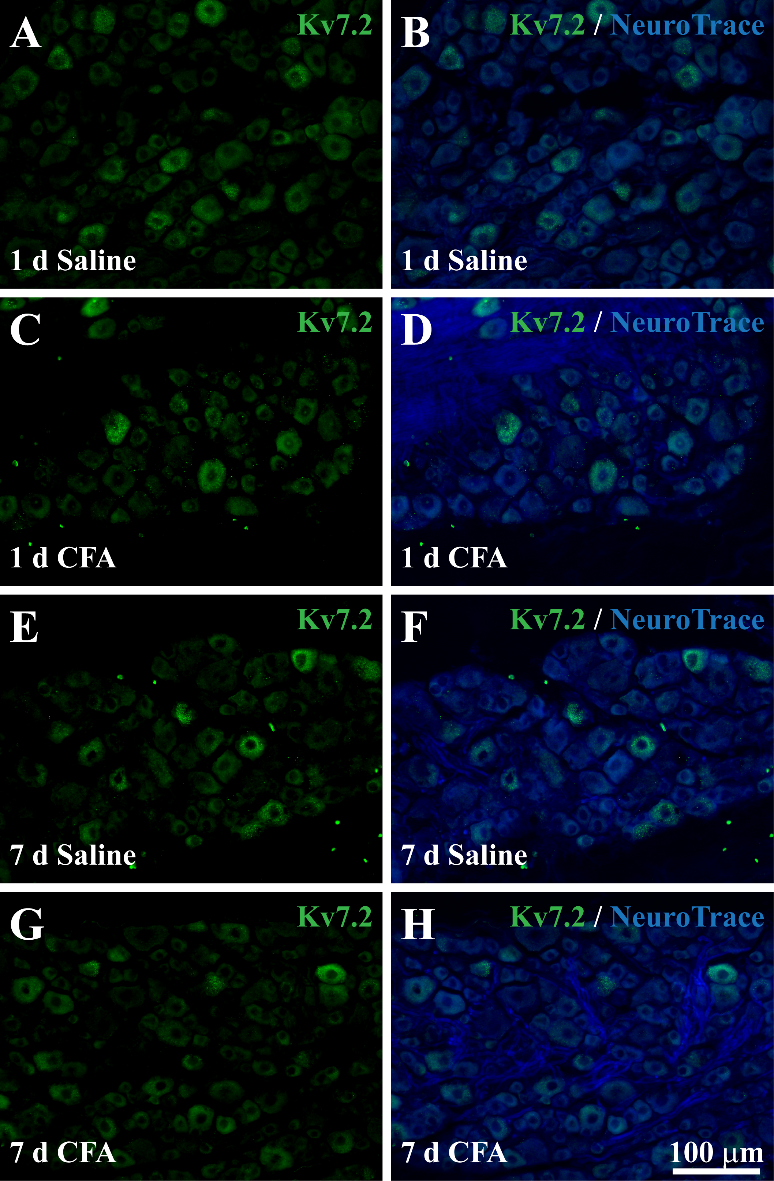


**Supplementary Figure 2.** Kv7.2 and NeuroTrace staining within DRG 1 and 7 days after injection of saline **(A, B, E and F)** or CFA. **(C, D, G and H)**. CFA did not produce changes in the percentage of Kv7.2(+) neurons. Scale in (H) applies to all images.


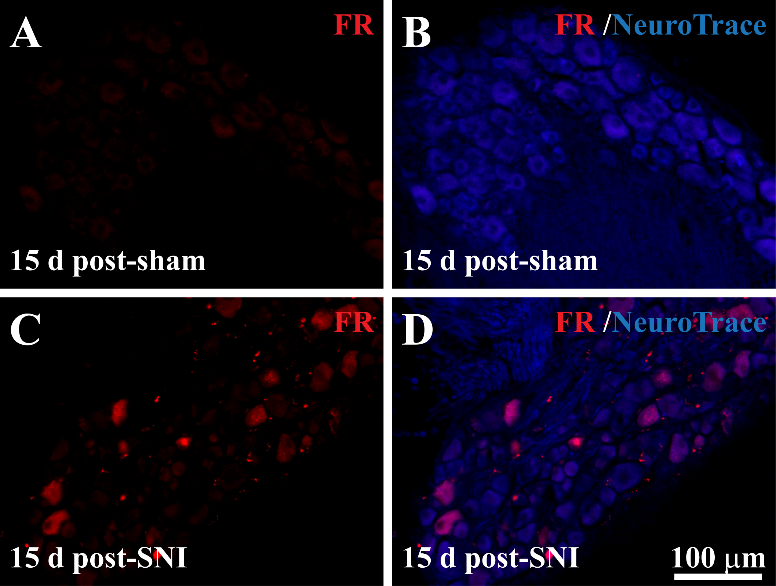


**Supplementary Figure 3.** Cryostat sections of NeuroTrace labeled DRG obtained from mice subjected to sham surgery (A and B) and SNI (C and D). Fluoro-ruby was applied by the nerve at the time of surgery. Note the absence of Fluoro-ruby tracing in sham operated animals (A) in contrast with SNI animals (C).


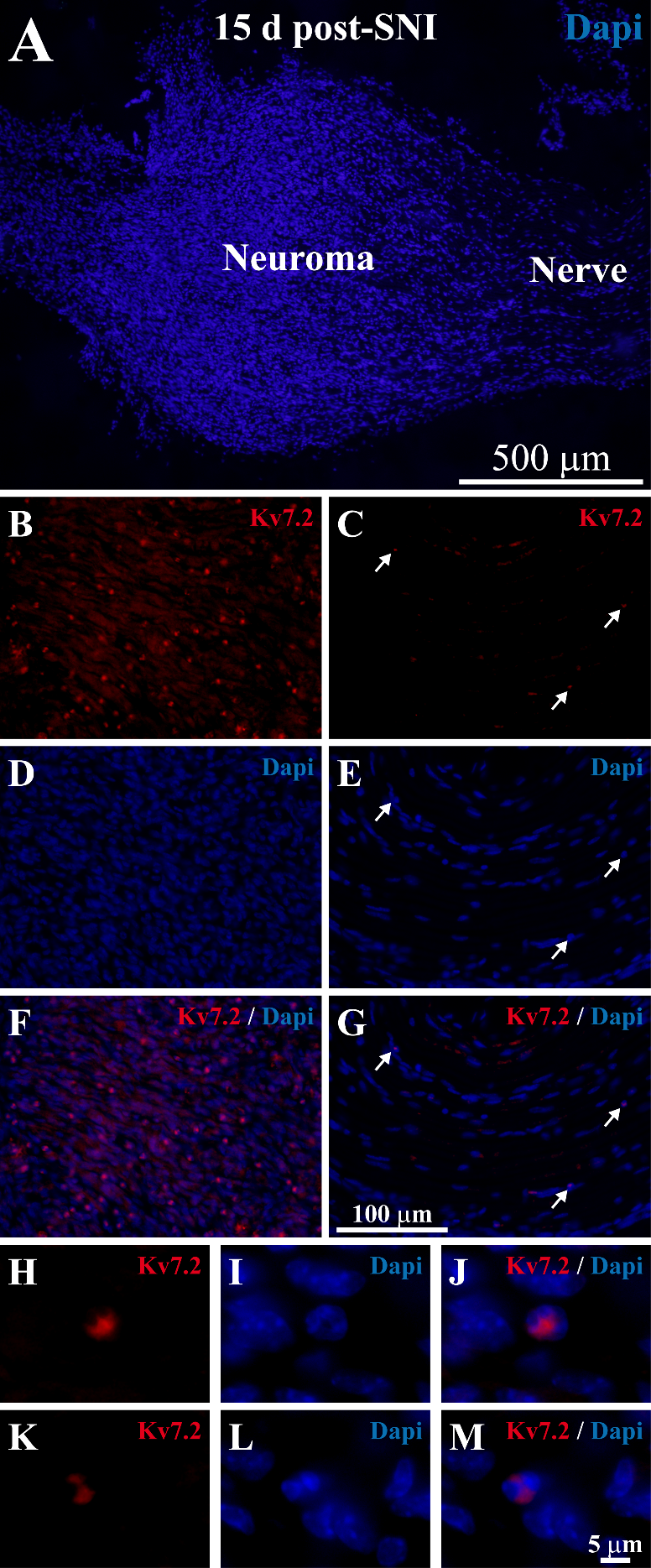


**Supplementary Figure 4.** Cryostat sections from sciatic nerves 15 days post-SNI were stained with Kv7.2 (red) and counterstained with Dapi lo label the nuclei (blue). **(A)** Low magnification image showing the nerve trunk and the neuroma, where the nuclei are more abundant and disorganized. **(B, D and F)** correspond to a magnification of the neuroma, where Kv7.2(+) aberrant structures are abundant. **(C, D and G)** are magnifications of nerve trunk. In this later region Kv7(+) structures are barely detected (white arrows). **(H-M)** are detailed views of Kv7(+) structures and the nuclei that surround them. Scale bar in (G) applies to (B-F) images. Scale bar in (M) applies to (H-L).


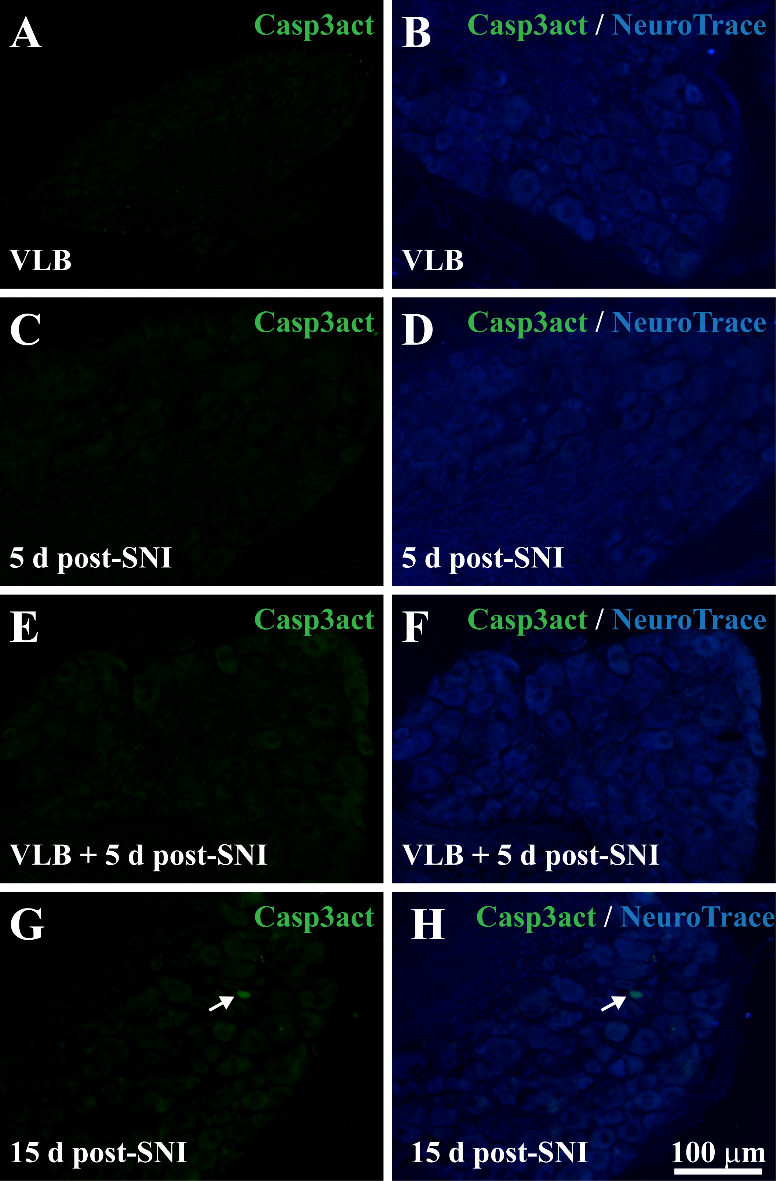


**Supplementary Figure 5:** Test of the toxic effect of VLB in sensory neurons. Cryostat sections from mice subjected to different treatments (VLB, SNI, VLB+SNI) were stained with activated Casp3 antibody (green) and counterstained with NeuroTrace (blue). Activations of Casp3 was not detected in VLB, 5 days post-SNI, or VLB + 5 days post-SNI animals (at least 500 neurons were analyzed in each DRG; n = 3 animals each condition). DRG from 15 d post-SNI animals were used as a positive control of Active Casp3 staining.


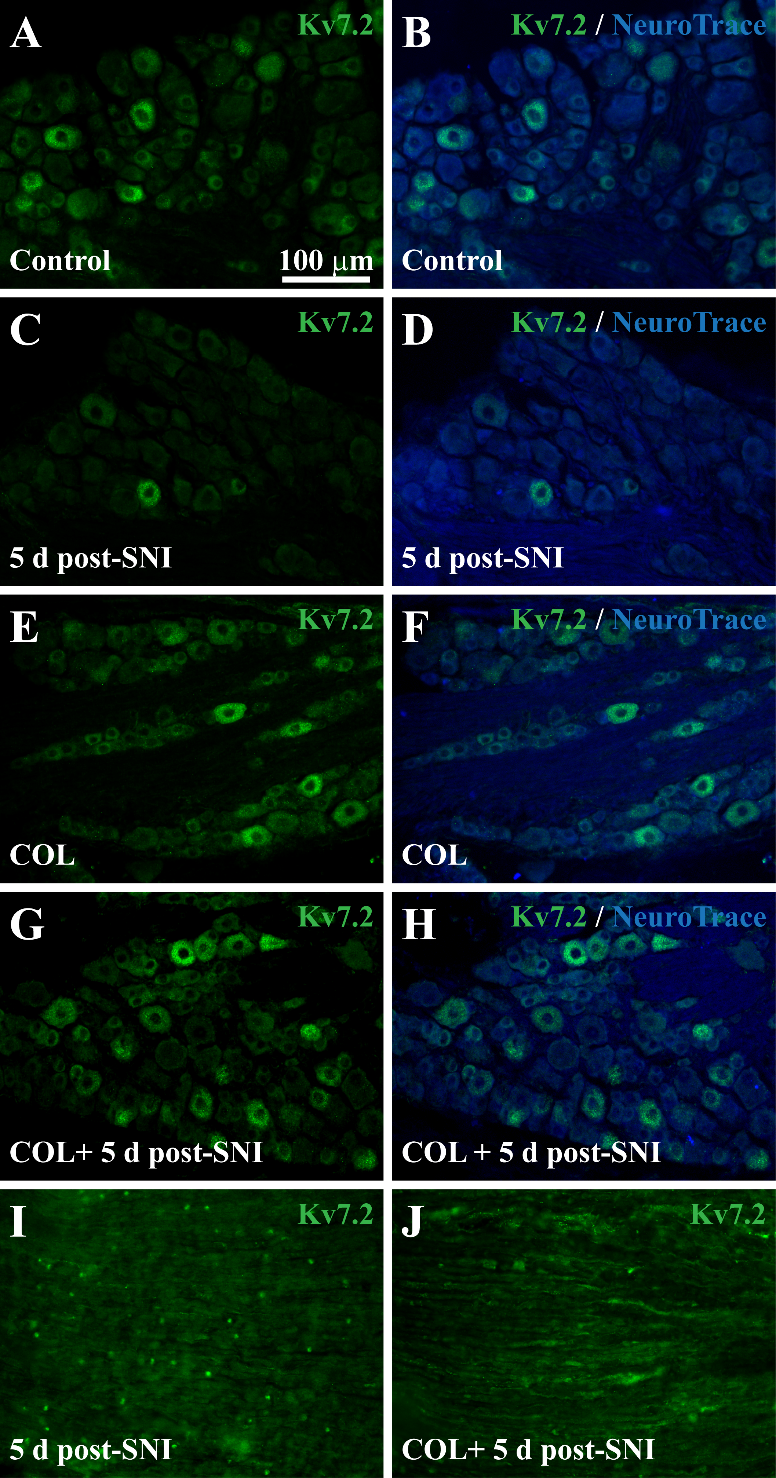


**Supplementary Figure 6:** Kv7.2 expression within the DRG and neuromas after blockade of the axonal transport with COL. **(A-H)** DRG from Control, 5 days post-SNI, COL and COL + 5 days post-SNI mice were stained with Kv7.2 and counterstained with NeuroTrace. Kv7.2 levels decrease within the DRG 5 days after SNI. COL treatment previously to surgery and during neuroma development prevents the reduction of Kv7.2 levels. **(I)** 5 days after surgery Kv7.2 accumulations are present in the neuroma. **(J)** When daily COL is administered during neuroma development, Kv7.2(+) structures are virtually absent in neuromatose endings. These results are similar to those obtained with VLB. Scale bar in (A) applies to all images.
